# Coxsackievirus A24 variant whole genome sequencing from clinical samples using a three overlapping amplicons strategy

**DOI:** 10.12688/wellcomeopenres.24183.2

**Published:** 2025-10-06

**Authors:** John Mwita Morobe, Samuel Odoyo, Arnold W. Lambisia, Edidah Moraa, Charlotte J. Houldcroft, Edward C. Holmes, George Githinji, Charles N. Agoti

**Affiliations:** 1 Kenya Medical Research Institute (KEMRI)-Wellcome Trust Research Programme (KWTRP), Kilifi, Kilifi County, Kenya; 2 Department of Genetics, University of Cambridge, Cambridge, England, UK; 3 School of Medical Sciences, The University of Sydney, Sydney, New South Wales, Australia; 4 Department of Biochemistry and Biotechnology, Pwani University, kilifi, Kenya; 5 School of Public Health, Pwani University, Kilifi, Kilifi County, Kenya

**Keywords:** Coxsackievirus A24 variant, Acute Hemorrhagic Conjunctivitis Kenya, Next generation sequencing

## Abstract

In January 2024, the Kenya Ministry of Health issued an outbreak alert following a surge in acute hemorrhagic conjunctivitis (AHC) cases along the Kenyan coast. Our investigations identified coxsackievirus A24 variant (CV-A24v) as the causative agent. In this study, we developed a novel whole genome sequencing assay for CV-A24v, and used it to recover three near complete genomes from the 2024 AHC outbreak in Kenya. This method will support future studies on CV-A24v genomic epidemiology and evolution across Kenya and beyond.

## Plain Language Summary

In January 2024, an outbreak of acute hemorrhagic conjunctivitis (AHC) along the Kenyan coast prompted an alert from the Ministry of Health. AHC is characterized by the sudden onset of painful, swollen, and red eyes, often with subconjunctival haemorrhage and a foreign body sensation. Our investigations identified coxsackievirus A24 variant (CA24v) as the causative agent. To characterize the diversity of the circulating strains, we developed a whole-genome sequencing protocol. This approach leverages tiled amplicon sequencing, wherein the viral genome is amplified in overlapping amplicons and subsequently reassembled to generate consensus sequences. We successfully recovered near-complete genomes from three clinical samples collected during the outbreak. This protocol enables rapid and cost-effective genomic characterization of CA24v, particularly in resource-limited settings. Our protocol lays the groundwork for improved surveillance of CA24v in Kenya and beyond.

## Introduction

Coxsackievirus A24 variant (CV-A24v) is a member of species *Enterovirus coxsackiepol,* genus *Enterovirus*, family *Picornaviridae,* and a leading cause of acute haemorrhagic conjunctivitis (AHC) outbreaks in the tropics, also referred to as "red eye or pink eye" disease ^1– 4^. The CV-A24v genome comprises a single-stranded, positive-sense RNA molecule of approximately 7,400 bp in length and encodes 4 structural proteins (VP4, VP2, VP3, and VP1) and 7 non-structural proteins (2A-2C and 3A-3D). To date, eight genotypes of CV-A24v (GI–GVIII) have been described, based on sequence homology within the VP1 gene ^5^.

As of 25 ^th^ April 2025, fewer than 119 complete or near complete genomes (>90% coverage) of CV-A24v were publicly available in GenBank database, these sampled between 1952 and 2024 from 25 countries. This is a relatively small number compared to the number of complete genomes for other outbreak viruses e.g., influenza A (~165,000 genomes), monkeypox virus (~8,500 genomes), SARS-CoV-2 (~17,000,000 genomes) and Ebola virus (~3,400 genomes). Even among *Enterovirus* genus, CV-A24v remains poorly represented. For example, there are approximately ~1,875 complete genomes for enterovirus A71, 1,657 for enterovirus D68, and 347 for poliovirus type 1. The small number of publicly available CV-A24v genomes may be in part explained by its limited availability of diagnostic capacity during outbreaks, sporadic nature of the infection, self-limiting nature of the AHC condition, and absence of a specific simple low cost genome sequencing methods ^6, 7^.

This paucity of CV-A24v genomic data limits our understanding of CV-A24v diversity, evolution and epidemiology ^7^. Previous efforts to generate CV-A24v genomes have relied on metagenomic sequencing and primer walking approaches ^2, 6^. However, these approaches are relatively expensive, technically more demanding, and require significant hands-on time in the laboratory ^8^. An alternative approach is an overlapping amplicon sequencing strategy in which the CV-A24v genome is amplified as series of tiled fragments which are then sequenced and reassembled ^9^. This strategy has been successfully used on several viral pathogens, including enteroviruses such a rhinovirus A15 and A105 ^10^, enterovirus D68 ^11^, echovirus 30 ^12^ and coxsackievirus B5 ^13^. Herein, we present a tiled amplicon approach for CV-A24v sequencing, developed in response to the 2024 AHC outbreak in coastal Kenya ^14, 15^, to enable high-throughput recovery of CV-A24v viral genomes directly from clinical samples and support future real-time genomic surveillance.

## Methods

**Figure 1.**
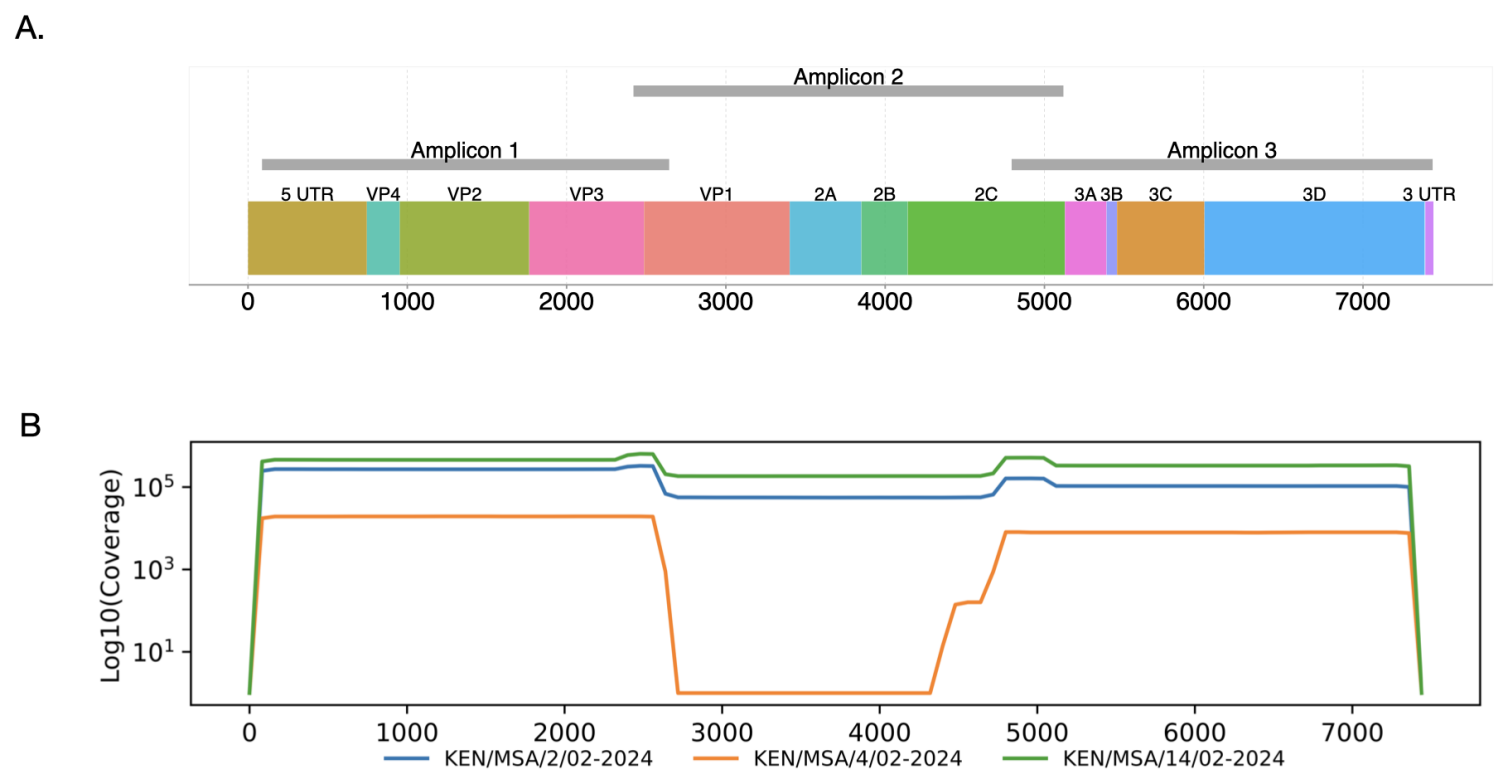
Genome maps. ( **A**) Schematic representation showing the position of overlapping amplicons in the CV-A24v genome (GenBank accession number PP548240). ( **B**) Coverage plots for KEN/MSA/2/02-2024, KEN/MSA/4/02-2024 and KEN/MSA/14/02-2024.

We used the *Primal scheme* algorithm with default parameters ^9^ and identified 12 primers (six pairs) that could bind to various positions within the CV-A24v genome. The input alignment utilized all the available 119 CV-A24v genomes (>95% coverage) in GenBank as of August 2024. This selection of primers aimed to have pairs that produce an amplicon size of ~2500 nucleotides. Laboratory optimization involved iterative testing of the primer pairs to evaluate their amplification success and coverage performance across a representative panel of CV-A24v-positive samples including the three isolates from Mombasa outbreak described below. The final three primers pairs were selected based on the following criteria: i) consistent amplification success across CV-A24v isolates, (ii) expected amplicon size and (iii) primer-pair positioning to maximize genome coverage. These primers pairs resulted in a three overlapping amplicons ( Table 1; Figure 1A). The resultant amplicons had overlapping regions of 222 nt between amplicon 1 and 2, and 325 nt between amplicon 2 and 3 ( Table 1; Figure 1A). This set was used to amplify viral RNA extracted from three CV-A24v positive ocular samples identified in early February 2024 on the Kenyan Coast ^14^, that had a diagnostic cycle threshold (Ct) of 32.29, 38.43, and 37.65 following qPCR.

**Table 1.** Characteristics of the six primers optimised for CA24 whole genome amplification and the PCR thermocycling conditions.

Amplicon Name	Primer Name	Strand	Melting Temperature (Tm) °C	Position Covering the Reference (PP548240)	Sequence (5'-3')	Product Size	PCR Cycle Condition
Amplicon 1	CVA24v_ amp1_F	+	63	88	ATACCCCTTCCCCACGTAACTT	2577	Initial denaturation 98°C, 30 seconds Denaturation: 98°C, 15 seconds Annealing: 59°C, 30 seconds Extension: 72°C, 1 minute Final extension 72°C for 2 minutes Hold at 4°C Number of cycles: 35
	CVA24v_ amp1_R	-	63	2644	CCAGATGCACCGGTCTCTAC		
Amplicon 2	CVA24v_ amp2_F	+	58.4	2420	TTTTAGTGTGCGTTTATTGAGAGACAC	2700	Initial denaturation 98°C, 30 seconds Denaturation: 98°C, 15 seconds Annealing: 60°C, 30 seconds Extension: 72°C, 1 minute Final extension 72°C for 2 minutes Hold at 4°C Number of cycles: 35
	CVA24v_ amp2_R	-	58.7	5119	GCCTCCATACAATTCCCAATG		
Amplicon 3	CVA24v_ amp3_F	+	59.5	2644	GTATTGGCCTCAACAAACTCACA	2643	Initial denaturation 98°C, 30 seconds Denaturation: 98°C, 15 seconds Annealing: 59°C, 30 seconds Extension: 72°C, 1 minute Final extension 72°C for 2 minutes Hold at 4°C Number of cycles: 35
	CVA24v_ amp3_R	-	62.2	7437	CCCCTACAACAGTATAACCCAATCC		

Viral RNA was extracted from the three ocular samples using the QIAamp Viral RNA Mini Kit (Qiagen) and reverse transcribed using the LunaScript® RT SuperMix Kit (New England Biolabs). A negative control (NC) (nuclease-free water) was included during both the extraction and reverse transcription steps. The cDNA was then amplified in three reaction tubes with the Q5® Hot Start High-Fidelity 2Master Mix (NEB) using the newly designed and optimised CV-A24v primers and thermocycling conditions as shown in Table 1. The PCR products were loaded on a 1.5% agarose gel to confirm amplification before purification using Agencourt AMPure XP beads. Library preparation was performed using the Ligation Sequencing Kit (SQK-LSK114) and Native Barcoding Kit (NBD96), and sequencing performed on the Oxford Nanopore Technologies (ONT) GridION platform. Base calling was conducted using the Guppy v6.5.7 ( https://github.com/artic-network/artic-ncov2019) under high-accuracy mode.

Genome assembly was performed using a sub-workflow of an in-house pipeline named " *ViralPhyl*" and available on GitHub ( https://github.com/kwtrp-peo/viralphyl). Base-called reads were demultiplexed using the ARTIC Guppyplex tool v1.6.2 with default parameters, applying a minimum Q score of 9. Reads shorter than 500 nt were filtered out using the toullingQC module. Consensus sequences were generated by aligning the reads to a reference sequence (in this case CVA24_2400060741_FRA24, GenBank accession PP548240). The reference strain was selected based on preliminary BLAST searches and phylogenetic analyses of partial VP4/VP2 sequences, which showed that our outbreak sequences clustered closely with CVA24_2400060741_FRA24, making it the most suitable reference for genome assembly. The reads were aligned using MiniMap2-v2.30 ^16^. A comprehensive list of tools utilized in viralphyl is available here: https://github.com/kwtrp-peo/viralphyl/blob/main/CITATIONS.md. Positions with genome coverage below 20 reads were masked with 'N'. The resulting consensus sequences were further refined using Medaka v 2.0.1 to correct potential sequencing errors. The recovered genome sequences were combined with publicly available CA24 genomes and aligned using MAFFT v7.5201 ^17^. A maximum likelihood (ML) phylogenetic tree was inferred using IQ-TREE v2.1.3 ( http://www.iqtree.org/) applying the GTR substitution model, with branch support assessed using 1000 bootstrap iterations. Nucleotide and amino acid variations between the newly sequenced genomes were analyzed using Snipit v1.6 ^18^. The analysis was performed with input options --sequence-type nt for nucleotide variation and --sequence-type aa for amino acid variation.

## Results and discussion

**Figure 2.**
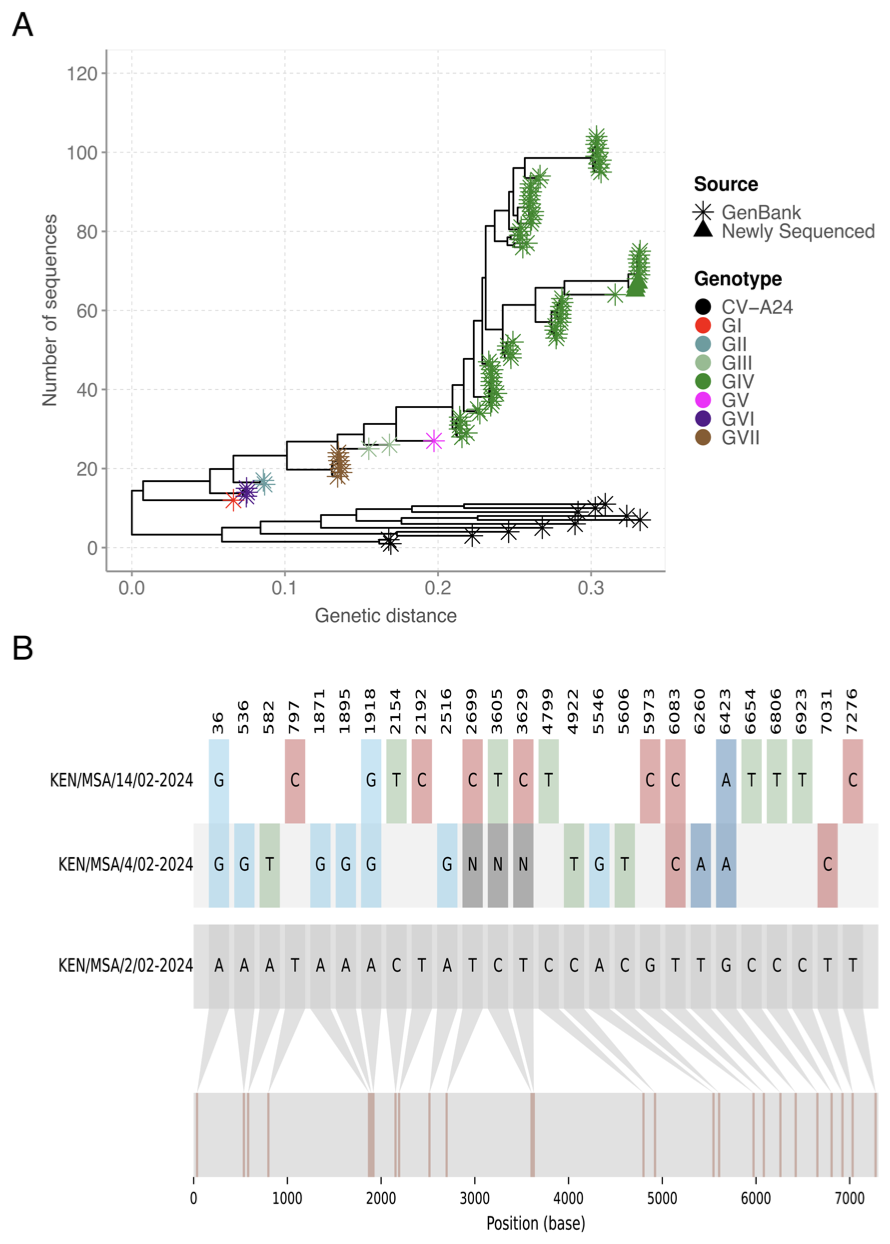
( **A**) Maximum likelihood phylogenetic tree based on the genome sequences of CV-A24v from this study (n=3) and previous outbreaks (n=119). ( **B**) Nucleotide alignment showing the nucleotide variations across the three CV-A24v genomes, with KEN/MSA/2/02-2024 as the reference sequence.

Two of the recovered genome sequences (KEN/MSA/2/02-2024 and KEN/MSA/14/02-2024) were 7,304 nucleotides (nt) in length ( Table 2), comprising the 5′ untranslated region (UTR) of 637 nt, complete open reading frame (ORF) of 6,645 nt, and 3′-UTR of 22 nt. Sequence KEN/MSA/4/02-2024 contained a 2,191-nucleotide gap within the VP1, 2A and 2B regions of the ORF due to amplicon 2 dropout ( Table 2, Figure 1B), likely due to low viral load, as indicated by a high Ct value > 38.43 in this sample. The new genomes generated here were classified as genotype IV, and clustered in clade comprising sequences sampled in Mayotte, an overseas department and region of France in February 2024 ( Figure 2A). The recovered genomes displayed nucleotide variations (n=25) across the entire genome ( Figure 2B). However, only synonymous mutations were observed (i.e., no amino acid substitutions) indicating conservation at the protein level despite genetic diversity.

**Table 2.** Genome length and coverage of the sequenced samples, along with their GenBank and SRA accession numbers.

Sequence	Number of Reads	Sequence Length	Coverage (%) Relative to Reference Sequence (accession PP548240)	GenBank Accession Number	SRA Accession Number
KEN/MSA/2/02-2024	8923	7304	98.13	PQ683184	SRX26951182
KEN/MSA/4/02-2024	1151	5113	70.00	PQ683185	SRX26951184
KEN/MSA/14/02-2024	16739	7304	98.13	PQ683186	SRX26951185
NC	29	-	-	-	-

This sequencing assay has some limitations. First, the selected primers did not capture the entire 5' and 3' UTR regions because the primers bind to regions within the UTRs, rather than at the terminal ends. Second, substantial genetic diversity exists within CV-A24v, yet our primers have only been tested with genotype IV which is the most commonly detected in recent studies. Subsequent testing of the novel method and primers described herein produced over 100 CV-A24v GIV genomes from coastal Kenya, all associated with the 2024 outbreak. Future testing of the method against diverse CV-A24v genotypes is needed to confirm similar performance across different genotypes.

In summary, we present a simple tiled-amplicon-based whole genome sequencing protocol for CV-A24v, that has great potential to support future studies on the genomic epidemiology of CV-A24v.

## Ethical approval

The samples analysed here were collected as part of Ministry of Health outbreak response activities to the AHC outbreak and as such written informed consent is not considered an essential step prior specimen collection. In such cases, individual consent is typically not required. Our analysis presented data that has been adequately anonymized as approved by the Institutional Review Board (IRB), allowing us to publish the outcomes of the outbreak investigations. The processing and sequencing of these samples was approved by the Institutional Review Board (IRB), including the waiver of individual consent given the public health emergency context. The molecular diagnostics and sequencing in scenarios of outbreak response by Kenya Medical Research Institute (KEMRI) - Wellcome Trust Research Programme (KWTRP) was approved by KEMRI Scientific Ethics Review Unit (SERU) Committee based in Nairobi, Kenya on May 19th, 2024 (Protocol #: KEMRI/SERU/CGMR-C/304/4894).

## Data Availability

The genome sequences reported in this work are available in GenBank under accessions PQ683184, PQ683185 and PQ683186. The raw sequencing reads are available in NCBI’s Sequence Read Archive (SRA) under BioProject accession PRJNA1193512. GenBank: Coxsackievirus A24 isolate KEN/MSA/2/02-2024 polyprotein gene, complete cds. Accession number PQ683184; https://www.ncbi.nlm.nih.gov/nuccore/PQ683184 ^19^. GenBank: Coxsackievirus A24 isolate KEN/MSA/4/02-2024 polyprotein gene, complete cds. Accession number PQ683185; https://www.ncbi.nlm.nih.gov/nuccore/PQ683185 ^20^. GenBank: Coxsackievirus A24 isolate KEN/MSA/14/02-2024 polyprotein gene, complete cds. Accession number PQ683186; https://www.ncbi.nlm.nih.gov/nuccore/PQ683186 ^21^. Sequence Read Archive: Genomic epidemiology of Coxsackievirus A24 in Coastal Kenya, 2024. BioProject accession PRJNA1193512; https://www.ncbi.nlm.nih.gov/nuccore/?term=PRJNA1193512 ^22^.
