# Supplementary material for: Coxsackievirus A24 variant whole genome sequencing from clinical samples using a three overlapping amplicons strategy
Source: Wellcome Open Res. 2025 Oct 6;10:316. Originally published 2025 Jun 10. [Version 2] doi: 10.12688/wellcomeopenres.24183.2 (PMC12569506; doi:10.12688/wellcomeopenres.24183.2)
Supplement: Peer review history

# Reviewer report: Reviewer response for version 2

**DOI:** 10.21956/wellcomeopenres.27491.r136393

**Published:** 2025-11-06

**Authors:** Jake D'Addiego

**Affiliations:** 1 UK Health Security Agency, Salisbury, UK

The manuscript is generally well written and provides a novel and cost-effective methodology for the targeted enrichment of CV-A24v nucleic acids, which will certainly aid in the recovery of genomic data from future CV-A24v outbreaks. Having read through the previous reviews, I think most concerns have been raised and/or addressed.  I do have some minor comments:

1. What is the prevalence of CV-A24v? How many cases of AHC are reported a year? A value might help highlight the public health significance of the presented methodology.  
2.  The authors use an alignment of 119 near complete genome sequences of CV-A24v. What is the genetic diversity within the alignment? Authors also state that there is substantial genetic diversity within CV-A24v; how much genetic diversity? Would be good to include a value.
3. Authors use the word “coverage” for two different metrics, one referring to % reference coverage indicating the proportion of the reference that was sequenced (in Table 2) and one referring to the number of times a specific base in the genome has been read, presented in the plot (Figure 1). I think using “depth” for the plot would be less confusing.
4. The sequencing depth/coverage for amplicon 1 and 3 (sample KEN/MSA/4/02-2024) seems to be quite high for the dropout of amplicon 2 to be solely attributable to low viral load. Furthermore, one of the other two samples (presumably KEN/MSA/2/02-2024) had a CT value of less than a cycle difference to the sample where the amplicon 2 dropout was reported (CT 37.65). Have the authors looked at primers mismatches using the recovered flanking regions to amplicon 2?
5.  Since a qPCR was utilised for CT determination, RNA copies/mL of sample should be determined and included in the table as the virus titres for the samples are currently not reported.
6. What was the final concentration of the primers in the PCR reactions? This value should also be included in the methods section.
7. In figure 2A it is very difficult to see the newly sequenced strains among the other GenBank sequences, as the branches are very close to each other and the shapes and colours overlap significantly. I would suggest either using a different software for phylogeny generation, or somehow better highlighting the newly generated sequences. Furthermore, despite 1000 bootstrap iterations were run for phylogeny generation, bootstrap values are missing from the figure. These should be included.  

Is the work clearly and accurately presented and does it cite the current literature?

Yes

If applicable, is the statistical analysis and its interpretation appropriate?

Not applicable

Are all the source data underlying the results available to ensure full reproducibility?

Yes

Is the study design appropriate and is the work technically sound?

Yes

Are the conclusions drawn adequately supported by the results?

Yes

Are sufficient details of methods and analysis provided to allow replication by others?

Partly

Reviewer Expertise:

molecular biology, genomics

I confirm that I have read this submission and believe that I have an appropriate level of expertise to confirm that it is of an acceptable scientific standard.

# Reviewer report: Reviewer response for version 2

**DOI:** 10.21956/wellcomeopenres.27491.r135507

**Published:** 2025-10-28

**Authors:** Charles Patrick Mcclure

**Affiliations:** 1 University of Nottingham, Nottingham, England, UK

Having read through the first round of reviews, the authors have endeavoured to answer and where appropriate rectify all issues raised. The reviews and amendments were all reasonable and sufficient for peer review and as such I don’t wish to unnecessarily burden the authors for this succinct and well written genomics-focussed short research note. However, I have spotted a few minor errors and as these will require amendment, I also have a few minor additional suggestions to make.

 The diagnostic assay will be of interest to some readers for confirmation of what may or may not detect this type that is relatively unusual in some regions. A reference or commercial assay name would suffice.

 Table 1 – reference coordinate for 3F presumably incorrect as product size does not add up and the number given is the same as the 1R coordinate?  It is positions 4795 to 4817 in the bedfile on GitHub.

 This highlights that the column title: *‘Position Covering the Reference (PP548240)’ *is not entirely correct  – the number given appears to be the position matching the 5’ end of each primer? But the primer of course covers a range of positions as per bedfile.

 I also note that the first primer in the github bedfile is not the same as in the table, rather it matches the very start of the reference sequence:

 PQ119796            1              23           CVA24_1_LEFT  1              +             TTAAAACAGCTCTGGGGTTGTTC

 This is also a different reference sequence to the table (PP548240).

 Ultimately, I would recommend unifying or at least clarifying any discrepancies between github and Table 1.

 Additionally, in the table title and page 5 in the text, two instances of updating CA24 to CV-A24 have been missed.

 Arguably if updating the table, the common cycle parameters might be better as a footnote or listing in the text as everything is the same apart from the annealing temperature (which isn’t particularly different) which would then stand out more.

*‘primers described herein produced over 100 CV-A24v GIV genomes from coastal Kenya, all associated with the 2024 outbreak’*

 I had wondered why these weren’t included, but I see from previous peer review that a manuscript in preparation will present these in due course. If no preprint is yet ready a ‘(manuscript in preparation)’ would be a helpful note to readers thinking the same?

Is the work clearly and accurately presented and does it cite the current literature?

Yes

If applicable, is the statistical analysis and its interpretation appropriate?

Not applicable

Are all the source data underlying the results available to ensure full reproducibility?

Yes

Is the study design appropriate and is the work technically sound?

Yes

Are the conclusions drawn adequately supported by the results?

Yes

Are sufficient details of methods and analysis provided to allow replication by others?

Yes

Reviewer Expertise:

Viral genomics, including amplicon WGS assay design and enteroviruses

I confirm that I have read this submission and believe that I have an appropriate level of expertise to confirm that it is of an acceptable scientific standard.

# Reviewer report: Reviewer response for version 2

**DOI:** 10.21956/wellcomeopenres.27491.r135120

**Published:** 2025-10-25

**Authors:** Sanjay Tikute

**Affiliations:** 1 ICMR- National Institute of Virology, Pune, India

The revised manuscript has been found with the clarifications incorporated and the changes as per the comments. It is updated and suitable, hence can proceed further for acceptance.

Is the work clearly and accurately presented and does it cite the current literature?

Yes

If applicable, is the statistical analysis and its interpretation appropriate?

I cannot comment. A qualified statistician is required.

Are all the source data underlying the results available to ensure full reproducibility?

Yes

Is the study design appropriate and is the work technically sound?

Partly

Are the conclusions drawn adequately supported by the results?

Yes

Are sufficient details of methods and analysis provided to allow replication by others?

Yes

Reviewer Expertise:

Virus isolation, characterization, diagnostics, animal experimentation, pathogeneses etc

I confirm that I have read this submission and believe that I have an appropriate level of expertise to confirm that it is of an acceptable scientific standard.

# Reviewer report: Reviewer response for version 1

**DOI:** 10.21956/wellcomeopenres.26675.r127366

**Published:** 2025-08-27

**Authors:** Sanjay Tikute

**Affiliations:** 1 ICMR- National Institute of Virology, Pune, India

Title: Coxsackievirus A 24 variant whole genome sequencing from clinical samples using a three overlapping amplicons strategy.

 Needs to revise the manuscript as per comments before indexing.

 Comments: The manuscript suggests the approach in overlapping the amplicon sequencing strategy, where genome is amplified as a series of tilted fragments which are then sequenced and reassembled. It’s a good methodology and technic in the field of molecular biology.

 Although the following minor comments has to be clarified.

 1. The short form of coxsackievirus A 24 should be rewrite as “CV-A24”. It should be changed in the entire manuscript.

 2. Clinical specimens (ocular samples) size is very low, needs to check with more numbers, (15 to 20 specimens or possible with the isolates) it will help to strengthen the method used.

 3. The Ct values of the samples ranges from 32.29 to 38.43, therefore it is required to select the samples with the lower Ct values and use for standardization. The value of positive control used is not mentioned.

 4. Specify the significance of the FRA24 strain used, mention it in the running text.

 5. Mention authors conflict of interest.

Is the work clearly and accurately presented and does it cite the current literature?

Yes

If applicable, is the statistical analysis and its interpretation appropriate?

I cannot comment. A qualified statistician is required.

Are all the source data underlying the results available to ensure full reproducibility?

Yes

Is the study design appropriate and is the work technically sound?

Partly

Are the conclusions drawn adequately supported by the results?

Yes

Are sufficient details of methods and analysis provided to allow replication by others?

Yes

Reviewer Expertise:

Virus isolation, characterization, diagnostics, animal experimentation, pathogeneses etc

I confirm that I have read this submission and believe that I have an appropriate level of expertise to confirm that it is of an acceptable scientific standard, however I have significant reservations, as outlined above.

# Response

**Published:** 2025-09-10

**Authors:** John Morobe

**Affiliations:** Kenya Medical Research Institute, Kenya

1. The short form of coxsackievirus A 24 should be rewrite as “CV-A24”. It should be changed in the entire manuscript.

**Our response: **Thank you for the correction. The abbreviation CA24v has now been changed to CV-A24 in the entire manuscript.  

1. Clinical specimens (ocular samples) size is very low, needs to check with more numbers, (15 to 20 specimens or possible with the isolates) it will help to strengthen the method used.

**Our response: **We acknowledge the limited number of ocular samples used in this study. These samples were collected as part of an emergency outbreak response, and the protocol was developed to facilitate rapid detection and sequencing. To validate its broader applicability, we have successfully applied the same protocol to a larger set of samples (stool and NP/OP) collected during the same outbreak. A separate manuscript describing these results is currently in preparation, and the resulting sequences have been deposited in GenBank (accession numbers: PX241976- PX242099).  

1. The Ct values of the samples ranges from 32.29 to 38.43, therefore it is required to select the samples with the lower Ct values and use for standardization. The value of positive control used is not mentioned.

Our response: While the Ct values of the ocular samples ranged from 32.29 to 38.43 (possibly due to collection during late phase of the infection), the protocol has proven effective in generating whole-genome sequences from samples (stool and NP/OP) across a wide range of Ct values (18-35). As indicated above were are preparing a follow-up manuscript describing these additional new genomes sequenced using this protocol.  

1. Specify the significance of the FRA24 strain used, mention it in the running text.

**Our response: **The FRA24 strain was selected as the reference strain based on BLAST searches and phylogenetic analyses of partial VP4/VP2 sequences from three representative samples compared with global CV-A24v sequences. These analyses showed that Coastal Kenya 2024 CV-A24 outbreak sequences were closely related to the FRA24 strain ^1^, making it the most ideal reference strain for our genome assembly protocol. This has now been explained in the revised manuscript.  

1.  Mention authors conflict of interest.

**Our response: **We confirm that there are no conflicts of interest to declare for all the co-authors, and we have now added this statement in the revised manuscript. **Reference**   1.           Lambisia AW, Morobe JM, Moraa E, et al. Identification of coxsackievirus A24 variant during an acute hemorrhagic conjunctivitis outbreak in coastal Kenya, 2024. *medRxiv*. Published online January 1, 2024:2024.12.04.24318475. doi:10.1101/2024.12.04.24318475

# Reviewer report: Reviewer response for version 1

**DOI:** 10.21956/wellcomeopenres.26675.r125724

**Published:** 2025-08-14

**Authors:** Peter van Heusden

**Affiliations:** 1 South African National Bioinformatics Institute, University of the Western Cape Faculty of Natural Science (Ringgold ID: 274799), Bellville, Western Cape, South Africa

While generally a well written paper presenting a useful technique, I have a few concerns:

 1. The "laboratory optimization" involved in selecting the final 6 primers was not explained. The sequence numbers used in the multiple sequence alignment used for primer design were also not specified.

 2. Details of base calling were omitted.

 3. The link https://github.com/kwtrp-peo/viralphyl leads to a 404 indicating either a missing or private Github repository.

 4. Versions of Guppyplex and MiniMap2 are omitted and Guppyplex is not cited.

 Most importantly, however, a BED file of the primers is not provided. PHA4GE provides draft guidance on what is expected in a primer scheme specification and at least the reference.fasta and primer.bed as per that guidance should be included: https://github.com/pha4ge/primer-schemes?tab=readme-ov-file#scheme-specification

Is the work clearly and accurately presented and does it cite the current literature?

Yes

If applicable, is the statistical analysis and its interpretation appropriate?

Not applicable

Are all the source data underlying the results available to ensure full reproducibility?

Partly

Is the study design appropriate and is the work technically sound?

Yes

Are the conclusions drawn adequately supported by the results?

Yes

Are sufficient details of methods and analysis provided to allow replication by others?

Partly

Reviewer Expertise:

Bioinformatics, especially of pathogens of human public health importance. I have recently engaged in research on Coxsackievirus A24v: https://www.medrxiv.org/content/10.1101/2025.04.28.24315795v1.

I confirm that I have read this submission and believe that I have an appropriate level of expertise to confirm that it is of an acceptable scientific standard, however I have significant reservations, as outlined above.

# Response

**Published:** 2025-09-10

**Authors:** John Morobe

**Affiliations:** Kenya Medical Research Institute, Kenya

1.  The "laboratory optimization" involved in selecting the final 6 primers was not explained. The sequence numbers used in the multiple sequence alignment used for primer design were also not specified.

**Our response:**  Primer design was performed using Primal Scheme inputting all 119 complete CV-A24v genome sequences available on GenBank database as of March 2024. This was following a multiple sequence alignment (MSA). Laboratory optimization involved iterative testing of various primer pairs to determine their target region amplification success across a panel of CV-A24v-positive samples with varied viral loads. The final three primers pairs selected were those showing:

- Consistent amplification across CV-A24v isolates.
- Expected amplicon size following gel electrophoresis.
- Primer-pair positioning to maximize genome coverage.

These details have now been added in the methods section of the manuscript.  

1. Details of base calling were omitted.

**Our response:** Thanks for pointing this out. In this protocol, basecalling  was automatically performed on the Oxford Nanopore GridION platform following sequencing using Guppy v6.5.7 under high-accuracy mode. These details have now been included in the Methods section of the updated manuscript.  

1. The link https://github.com/kwtrp-peo/viralphyl leads to a 404 indicating either a missing or private Github repository.

**Our response:** We apologize for the problem in accessing the link. The repository has now been made publicly accessible and the link is provided in the revised manuscript should work fine.  

1. Versions of Guppyplex and MiniMap2 are omitted and Guppyplex is not cited.

**Our response: ** The version of the tools have now been added to the Methods section. ARTIC  guppyplex v1.6.2 and Minimap2-v2.30 Additionally, full details of the tools used in the assembly and their respective versions are available on the ViralPhyl GitHub repository.  

1. Most importantly, however, a BED file of the primers is not provided. PHA4GE provides draft guidance on what is expected in a primer scheme specification and at least the reference.fasta and primer.bed as per that guidance should be included: https://github.com/pha4ge/primer-schemes?tab=readme-ov-file#scheme-specification

**Our response: **The BED file of the primers and reference used in assembly is provided on the viralphyl GitHub repository https://github.com/kwtrp-peo/viralphyl/tree/main/primerschemes/cva-24/V1.2.0
